# QUEER REMAINS: Derek Jarman’s Archives and Medieval Reliquaries, 1981–ca. 1993

**DOI:** 10.1215/10642684-10920606

**Published:** 2024-01-01

**Authors:** E. K. Myerson

***I*** can smell chlorine on the inside of my face mask, sitting in a back room at Tate Britain, nausea rising in my throat. I am trying to read the handwriting of the film-maker and artist Derek Jarman (1942–94), whose flowing script was rapidly deteriorating as he wrote in his hospital bed, stumbling and misspelling names, clumsily scratching over loose lined sheets. Jarman’s partner Keith Collins sat beside him, learning how to use an IV drip. “For years,” Jarman (2018a: 208) wrote, in 1989, “The Middle Ages have formed the paradise of my imagination.… It is something subterranean, like the seaweed and coral that floats in the arcades of a jewelled reliquary.” Like Jarman’s imagined reliquary, these archives are made of jewels and ashes: the aesthetic alongside bodily remains.

In this article I take a roughly chronological journey through Jarman’s archives, tracing the development of his imagination of medieval reliquary devotion as a practice of queer mourning and memorialization. My encounter with these archives gave me a sense of the loss that occurs when a queer artist is canonized by the mainstream establishment, moving them from the space of the community into the lineage of the nation. Instead, drawing on Jarman’s own devotional approach to the medieval past, I seek an alternative relationship with archival objects: a relation of communal mourning and queer veneration. I begin in 1981, with Jarman redrafting the scripts of his unrealized medieval film project *Bob-up-a-Down* as the AIDS crisis begins to act on queer lives and imaginations. The end of my project, ca. 1993, is determined by Jarman’s own illness, progressing toward his death from AIDS-related causes on February 19, 1994; his final papers are undated. In my research, I moved physically between archives at the British Film Institute (BFI), Tate Britain, the British Library, and the Wellcome Collection—all institutional repositories in London—to Jarman’s garden in Dungeness (Kent). Following his HIV diagnosis in 1986, Jarman moved to a former fisherman’s hut, Prospect Cottage, began to plant in the shingle, using medieval and early modern herbals as his guides, and created a sacred, memorializing space that remains a pilgrimage site for queers and horticulturalists today.

After Keith Collins’s death in 2018, Prospect Cottage and its contents were put up for sale, preserved for public ownership after a crowdfunding campaign led by Tilda Swinton, Jarman’s famous protégé. The arrival of Jarman’s papers in the Tate Britain repository witnesses the loss of not only their author but also their long-term custodian. I came to the Tate collections to find unique texts: aesthetic objects and unpublished, autograph writings, which have the aura of the nonreproducible (Benjamin 2002: 105). Preserved in files labeled “Bedroom,” “Painting Room,” and “Attic,” these records recall the intimate spaces in which they were recently kept. Despite now being housed inside dominant cultural institutions, Jarman’s archives maintain an affinity with ephemera, the constitutive elements of the queer counter-archive (Kumbier 2014: 69). Jack Halberstam (2005: 169) writes that the queer archive “has to become a floating signifier for the kinds of lives implied by the paper remnants of shows, clubs, events, and meetings.” These speculations are realized in the Jarman archives, in which the lives of Jarman and his lovers, friends, and audience float through the collections, evading containment.

Although Jarman as an artist has now been canonized by the establishment, he is also a queer saint whose canonization was enacted by politicized nuns in drag habits (Ellis 2009: 222). In 1991 Jarman was recognized as a saint by the international order of queer nuns, the Sisters of Perpetual Indulgence, in recognition of his many holy deeds as filmmaker and AIDS activist, in addition to his beautiful nose. Subtitling his polemical memoir *At Your Own Risk* “A Saint’s Testament,” Jarman (2019a) swiftly integrated his new sanctified authority into his self-conception. In 1981 the San Francisco nuns had created the order’s first official relics, gathering ashes from the site of the destroyed Barracks Bathhouse and placing them in vials that circulated as holy fragments (fig. 1). The sisters provided Jarman with a model of reliquary devotion in queer practice. The making of queer saints acts as an alternative to modern national canonization, which is enacted through official archives and aesthetic canons. National veneration is monolithic, affirming the status of the subject through their relation to the power of the state, raising that figure above the public. Queer sainthood is created through a communal, aesthetic ritual that makes sacred those whom the state has oppressed. Whereas the subjects of national veneration are highly resistant to accusations of their inauthenticity, queer saints are openly inauthentic in their formation as performative, anti-monumental icons.

After his death, Collins was instrumental to the preservation of Jarman’s legacy as saint, not least through his assemblage of two hundred “Reliquary” boxes, containing postcards, fragments of celluloid, poppy seeds, and fabric from Jarman’s saintly robe, which were sold for charity in 1996 (Mills 2018: 143–44). In medieval studies, Robert Mills’s (2018) work lays the critical foundations for study of Jarman’s sainthood. Conducting his research on-site at Prospect Cottage between 2014 and 2018, Mills’s access to the Jarman archives was mediated through Collins, and that personal contact leaves its trace on Mills’s research. In conversation with Collins, Mills learned that Jarman wore a reliquary on his person: a glass brooch filled with relic-like fragments, which Jarman had brought from Rome (144). Mills’s book provided me with a framework for understanding Jarman’s engagement with medieval religious imagery in the context of his critique of institutional Christianity. Building on Mills’s work, I offer a new perspective on Jarman’s reliquaries as both aesthetic and memorializing practice, analyzing new fragments of the Jarman archives in their recently altered contexts.

Like Halberstam’s archival “floating signifier,” Jarman’s premodern is uncontainable, natural, tangled: seaweed floating through a submerged reliquary. There is a rejection of stasis in Jarman’s approach to history. For Jim Ellis (2009: xii), Jarman’s premodern spaces are sites of queer liberation, configuring communal ways of being, in resistance to present-day conservative modes of existence. Extending Ellis’s argument, I contend that Jarman deploys the communal language and practice of medieval reliquary devotion in defiance of the politicized denial of ritual. As has been acknowledged but as bears repeating, the AIDS crisis produced an urgent need for new forms of grieving (Cvetkovich 2003: 156). Jarman’s friend, the critic Simon Watney (1988: 80), described how in Britain “the ‘AIDS victim’ must be publicly seen to be humiliated, thrown around in zip-up plastic bags, fumigated, denied burial, lest there be any acknowledgement of the slightest sense of *loss*.” Jarman created his works in this hostile context of disrupted mourning.

Just as I focus on “Jarman’s archives,” I focus here on “Jarman’s reliquaries.” Rather than a transhistorical comparative study, I consider medieval reliquaries as they appear through and to Jarman. A brief note on terminology: *relic* refers to a fragment of a saint’s body—stained earth, clotted blood, a fingernail—or to an object from a holy site, like a stone. In the medieval period, these fragments were subject to both theological debate and fervent veneration (Bynum 1991: 271). In that intense context, the reliquary becomes an essential aspect of medieval devotion: as Cynthia Hahn (2017: 11) writes, “The reliquary *makes* the relic.” In keeping with these medieval frameworks, Jarman’s reliquaries are performative artifacts. Certain significant subjects fall outside my present remit: I will not dwell in depth on the holy blood that reliquaries sometimes contained; Jarman’s response to medieval Passion devotion is a subject that I have considered elsewhere (Myerson 2023).

I bring “a queer medievalist’s touch” (Dinshaw 1999: 54) to these fragile materials, exploring the sensations of being touched by and touching the past medieval and modern. This research worked its way into my body, the skin of my latex-gloved hands growing dry in Tate Britain, as I tried not to cry, reading the hospital journals. Following Jarman’s lead through medieval and modern archives, these materials required me to take an affective methodology when approaching their contents. I felt an urgent need for a critical method that could witness my discomfort while still allowing me to interpret these collections as objects of cultural value.

## Seeking Reliquaries in Jarman’s Archives

I arrive at the BFI Reuben Library to read the unpublished drafts of Jarman’s unrealized medieval film project, *Bob-up-a-Down*. First developed as a script in 1979, in collaboration with Tim Sullivan, the film is firmly located in the medieval past, in the seaside village of Dancing Ledge (Peake 1999: 291). Sullivan’s early drafts of the script locate the action in an unspecific medieval time period, in a village dominated by intercommunal strife prompted by the forbidden love of Bob-up-a-Down (the hurdy gurdy player) for Prophesy (the granddaughter of the goose-woman). A brown cardboard folder in the BFI archives, labeled “Bob up a Down” in Jarman’s flowing handwriting, contains the second draft of the script, marked as “by Tim Sullivan / based on an idea by Derek Jarman” and dated February 24, 1981.^1^ In Sullivan’s direction: “The film opens to the whirring sound of a medieval hurdy-gurdy, playing a wedding song.”^2^ By 1983 the contexts for the historic narrative had altered—both within and outside the text. As HIV/AIDS began to dominate queer communal life, Jarman resituated *Bob-up-a-Down* in the context of the Black Death. Several pages of handwritten notes show Jarman’s engagement with medieval records while reworking the script. On one loose pink sheet, Jarman writes in the voice of a medieval plague survivor: “we fled to the remotest edge of the world.”^3^

These drafts of *Bob-up-a-Down* show Jarman’s first definite engagement with the image of the medieval reliquary, as a symbol situated in the context of plague. A new draft of the script, handwritten by Jarman and dated October 1983, sets the scene as follows:

England 1359 The beautifull Lady Angharad with four of her liege Lords travels along the priests way through the desolate isle of purbeck with its windswept downs and stormy coastline to Dancing Ledge; where fate has decided she will be immured for life as an anchoress on a bleak and rocky headland overlooking the ocean. In a jewelled relique she carries a precious thorn from the crown of thorns that has cost her a great part of her patrimony which had saved her and her bondsmen from the great plague that has swept through England Dancing Ledge to a little hamlet of 40 souls which lives by a meagre agriculture scratched from the forbiding [*sic*] landscape.^4^

Jarman here relocates *Bob-up-a-Down* from a generalized late medieval past, to the specific historic moment of the first years of the bubonic plague in England. Both the “great plague” and the “jewelled relique” are new additions to the text. Although the date is off by a decade (the bubonic plague arrived in England in 1348), the affective context is vivid. Lady Angharad’s enclosure is presented as not simply a response to a divine calling but a practical reaction to the dangers of human contact in a time of plague. Lady Angharad’s fate is more extreme than historically attested forms of medieval anchoritic enclosure; Julian of Norwich’s cell, attached to St. Julian’s Church at Norwich Priory, had semipublic areas into which visitors were able to enter (Salih 2009: 157). Jarman’s scene is immersed in an imagined medieval past, evoking illuminations of relic processions. Lady Angharad’s devotion to the Holy Thorn is both mystical and medical, the relic attributed with the power to ward off plague. The curative Passion relic is presented in relation to the specific calamity of a new pandemic—a historic medical context that lends increased urgency to the desire to find salvation. Jarman implicitly brings together the historic moment of medieval plague and his contemporary context of the AIDS crisis, but not through invoking societal moralism, the trope of disease as the product of sin, which recurs in medieval and modern discourse. Instead, Jarman invites a transhistorical dialogue predicated on a shared experience of suffering and the desire for a cure.

The BFI archives evidence Jarman’s sustained engagement with the historical realities of the bubonic plague, drawing an affective, transhistorical connection between the early 1980s and the late 1340s. In a separate, pink booklet of notes titled “Notes for Bob up & down,” Jarman wrote, and then erased, the date: “1348”—indicating that the 1359 dating error was a casual slip.^5^ Here Jarman assumes the voice of a learned eyewitness, writing:

on 20 march 1345 at 1. p.m. occurred a conjunction of saturn Jupiter and mars in the house of Aquarius and there rained a vast rain of fire … In this year appeared the Black Death which men called Foul Contagion sparing no man be he great or less … the pest-ilence betrayed itself by the emergence of tumours in the groin and armpits some as large as a common apple or egg … some were seized by the plague as they administered spiritual aid and often by a single touch or breath were plague stricken and perished before those they had come to assist.

oh happy posterity who will not look upon experience such abyssmal [*sic*] woe and will look upon our testimony as a fable.^6^

Jarman echoes the premodern explanation of plague outlined in the 1348 Paris Consilium, written by the medical faculty of the University of Paris (Horrox 1995: 159). The plague is attributed to astrological fate, an explanation that combines with the medieval vocabulary of “pestilence,” the emphasis on spiritual aid, to distinguish these events from those unfolding outside Jarman’s notebook. The Black Death and HIV/AIDS are not rendered interchangeable—the symptoms of medi-eval disease are clearly listed. The erasures show Jarman’s replacement of the postmedieval vocabulary of “plague” with the more authentic terminology of “pestilence.” Jarman’s identification with the horrors of the arrival of illness lends contemporary urgency to his notes—unpunctuated, flowing prose. The first-person voice gains momentum in this passage, stacked clauses echoing the pace of devastation and the scale of the tragedy. Moments that evoke the experience of the AIDS crisis gain additional pathos: feelings of “terror,” the deaths of “young and old alike.” The first-person plural situates the speaker in the midst of an embattled community, with the imagined address of the medieval survivor to “happy posterity.” Writing in the early years of the HIV/AIDS epidemic, Jarman layers the lines with devastating dramatic irony—speaking back from “posterity” to the present. Jarman’s empathic relation to the suffering of the past is clear. For Carolyn Dinshaw (1999: 173), the queer touch of the past is a political project, particularly valent in the context of AIDS, in which touching the sick was made taboo. Articulating a practice of queer historic touch, Jarman invites us to follow him, to bring the bodies of both the recent and the distant past into contact with our own.

In Jarman’s incomplete redraft of *Bob-up-a-Down*, written with this heightened awareness of plague, Lady Angharad’s reliquary becomes a prominent image, in another new scene:

The Lady angharad holds the jewelled relique against the sun deep in prayer at the very edge of the rocky cliff facing the sea. The silence is brocken [sic] only by the mewing of the sea gulls and the clatter of stones as the villagers creep up hidden by the huge boulders to watch their strange visitors

Prophesy more curious than the rest comes out of hiding into the open and watches the penitents on their knees

angharad kisses the relique with the thorn and prays

angharad

I pray god grant me

whan I have ended all mine

adversite graunt me in

paradise to have a mansion.^7^

The scene of relic devotion is an addition to the earlier text, echoing the relic procession with which this revised version opens. Lady Angharad’s speech is excerpted from an anonymous fifteenth-century lyric, “Farewell this world!,” which was printed in R. T. Davies’s (1966: 206–7) *Medieval English Lyrics*, an edition of which was kept in Jarman’s library. Jarman places the lyric in a context of plague-threatened mortality. The reliquary appears as an object that demands a ritual, an act of communal public devotion.

In their fragmentary nature, these papers in the BFI archives demand an affective critical response—both devotion and reconstruction. Jarman’s recurrent image of the medieval reliquary provides an inscribed critical response. The visitor to a shrine is already taking part in a ceremony—a pilgrimage. The reliquary relies on that communal ritual performance, without which the relic ceases to function as sacred; its status is dependent on devotional reiteration. In addition, an unfinished text requires imaginative reconstruction: without the reader’s imagination, these scenes can barely be visualized. Attending to the fragments of these scripts is my way of attempting critical restoration.

## Visionary Fragments

In their form and in their contents, Jarman’s archives self-articulate as both shrine and debris. For me, the image of wrecked divinity describes the collections: the traces of a queer saint. Using this religious language, I signal my desire for membership in the community formed around Jarman’s body by the Sisters of Perpetual Indulgence, not in a formal sense but as an affective relation. In archival practices of martyrology, the discarded body is remembered by the community to which that saint belonged. There is an affinity between this view of Jarman’s archives and Walter Benjamin’s understanding of the archive as an archaeological site, in which history can be activated through traces (Russell 2018: 113). In fragments, Jarman’s archives preserve himself in order that his works can be activated through research and other rituals.

Staying with Jarman’s premodern referents, the image of sacred debris was central to medieval theology. Contemplation of the broken contents of shrines was considered the source of insight. Meditating on the Passion, the fourteenth-century anchorite and mystic Julian of Norwich received the following vision:

One tyme mine understondyng was led downe into the see-grounde, and there I saw hill and dalis grene, semand as it were mosse begrowne, with wrekke and with gravel. (Julian 2015: 45)

One time my understanding was led down into the sea-ground, and there I saw hill and dales green, seeming as it were overgrown by moss, with wrekke and with gravel.^8^

The ocean floor—prefiguring submarine travel—appears verdant, far from natural light but recalling rolling hills. The word *wrekke* here can be interpreted as meaning either “seaweed” or “wreckage”—an apt ambiguity, present too in Jarman’s weed-strewn reliquary, an emblem of misplaced treasure. Julian (2015: 45) interprets her underwater insight as signifying “that if a man or a woman were under the broade watyr, if he might have sight of God … he should be save in body and soule and take no harme.” In her gloss, the “broad water” is read in line with biblical imagery of floods and storms, as a mortal danger from which only faith can provide salvation. The sight of God is witnessed in the marvelously green seabed—Julian gains assurance of divine presence through the vision of moss, seaweed/wreckage, and sand. Julian’s visionary gaze pans the ocean floor like a film camera. Jarman was a devoted reader of Julian. On December 19, 1989, he noted in his diary: “Read Julian of Norwich’s *Revelation of Love* to the gentle whirr of the washing machine” (Jarman 2018a: 208). This allusion precedes Jarman’s aforementioned image of the submerged reliquary: Julian was foundational to Jarman’s understanding of the visionary potential of medieval fragments.

Jarman’s multiple, incomplete revisions of *Bob-up-a-Down* show his development of the figure of Lady Angharad, in relation to these ideas of destruction and vision. In these scenes, the sacred aura of Angharad’s reliquary appears in a context of incomplete creation, an unfixed image. On the first page of the booklet “Notes for Bob up & down,” Jarman writes:

10 scenes: *super 8 vision.

vision Jerusalem

death of knight on sea shore

saints tears

the battle of caster and sent with nativity play

travelling gypsies

the finale

the blind leading the blind

the castle of death

wolves in forest eating body very easy

the Travelling princess

children in the armour

the rape scene

the princess in her palquin dies.^9^

These fragmentary notes foreground a motif from John’s *Revelation*, the vision of the New Jerusalem. Mills (2022) has explored the influence of the book of Revelation on Jarman’s apocalyptic imagination. Here Jerusalem appears amidst a collage of motifs from romance and modern medieval film. Jodi-Anne George (2019: 200) has commented on Jarman’s integration of the experimental “trance film” genre with the traditions of medieval dream vision poetry, in his realized films *The Last of England* (1987) and *The Garden* (1990). These planned notes for *Bob-up-a-Down* imply a comparable treatment in this unmade project, employing the home film aesthetics of Super-8 as a way of evoking premodern visionary experience. Presented in list format, the disjuncture between the proposed scenes is heightened—the reader enters into a dreamlike state of associative thinking in order to follow the imagery. Although there is violence, there is humor too in these notes, as in the casual “very easy” consumption of the unidentified body by wolves. The context of plague is invoked in the “castle of death,” while the “saints tears” evoke established medieval iconography. On the next page of the booklet, Jarman ambiguously contextualizes these scenes: “The anchoress lives alone with her crow to whom she speaks and with which she has her visions of a world outside the cairn … The castle is devastated by plague. The bishop sends the lady to do penance. penance scene. / the hermitess is turned into a saint / but the black death has caught up. They all die Lady Bishop and villagers for not saving her.”^10^ In light of this synopsis, the previous list of Super-8 visions can be interpreted as Lady Angharad’s prophetic visions from within her cairn—placing the viewer within the state of enclosure, imaginatively accessing the anchoress’ revelations.

Later on in the same pink booklet, Jarman sketches out a variant version of the narrative, with a new emphasis:

Prologue is

1. Black Death.

anchoress lives in a well very vain and goes down to sea shes [sic] the richest lady in the village, a great collector like a magpie mentality of 12….

2. Lady Belle Sans pareil

Very chivalrous with her knight completely in love. she dies from plague after anchoress has given him a talisman

3. he no longer believes in god he batters her door down trying to keep him out

--> he ravages the bejewelled hermitess.

meanwhile dance of death in castle the [sic] go to anchoress like blind following

she is dying outside in the light

The Bishop incites the world against the Knight he is the devil. The kids

track him down to beach

Kill him with stone on the beach

in final scene we see the whole world at the shrine of the new saint.^11^

In this radically revised version of *Bob-up-a-Down*, plague is the driving force of the film, and the tragic love story between Bob and Prophesy has been excised altogether, replaced by the elite romance of Lady Belle Sans Pareil and the unnamed knight. Whereas in the incomplete, surviving 1983 draft, Lady Angharad travels to Dancing Ledge to escape the plague, in this planned version she is not only unable to escape infection but also becomes herself the vector of disease, bringing the plague to the community and transmitting the disease through the distribution of talismans. Lady Angharad’s reliquary is therefore an ambivalent signifier in this narrative—a harbinger of death where it promised cure. There is no miracle offered to provide hagiographic rationale for the sanctification of Lady Angharad, although the assault of the anchoress gives an image of martyrdom. The knight, who responds to her role in spreading the disease with violence, is figured as the devil, while the sick woman remains holy. Another note in Jarman’s pink booklet, headed “THIS IS IT,” reads, “maybe we tell the story of the anchoress more at the beginning—we need to establish the Anchoress as having magical powers in sickness.”^12^ The phrase “in sickness” preserves an ambiguity as to whether her magical powers are focused around the curing of others’ disease, or if the occasion of her own sickness is the cause of her powers. In either case, there is a mystical relationship between healing and disease established in this unfinished script, and it pivots around the person of the noble visionary anchoress. Lady Angharad is doubly connected to relic devotion: first, in bringing the holy thorn to her enclosure, and second, in herself becoming sanctified and enshrined, the focus of global communal veneration. In this version of *Bob-up-a-Down*, Jarman offers a vision of a saint who has been the transmitter of plague—her body both infectious and salvific, assaulted and transcendent. Written against the backdrop of HIV/ AIDS, in which public ceremonies for those who died from AIDS-related causes were interrupted and repressed, Jarman’s imagination of the “whole world” at Lady Angharad’s shrine foregrounds communal veneration of the sick anchoress, whose visions are inextricable from her experience of disease.

There is an inescapable resonance between the fictional Lady Angharad, imagined as moving to a remote coastal shrine in a time of terrible plague, and Derek Jarman’s move to Prospect Cottage in his own context of HIV/AIDS. To read the 1983 scripts as autobiographical would be anachronistic: Jarman had not even visited Dungeness at the time of the composition of those texts. However, *Bob-up-a-Down* remained in Jarman’s mind in his own response to his new environment. Writing in 1987, while planning out a new film project—which eventually would become *The Garden* (1990)—Jarman speculated, “we could build the cairn from Bob-up-a-Down into this project so Tilda becomes Prophesy” (Peake 1999: 397). Jarman was reluctant to let the abandoned project go. The cairn never did make it into his film—nor was Tilda Swinton cast as Prophesy, instead taking on a series of biblical roles in a reimagined dream vision retelling of the Passion. The medievalist narrative that Jarman and Sullivan constructed remained unrealized. However, fragments of the text recur: in one project book for *The Garden*, now held in the Tate collections, entitled “In Borrowed Time,” Jarman copied out the lyric “Farewell this world,” previously used as Lady Angharad’s prayer, now relabeled simply and inaccurately as “14th Century Poem.”^13^ In its later context, the poem appears amidst Jarman’s reflections on his garden: the coastal edgeland that Jarman planted as a living memorial and healing space.

The visionary fragments of *Bob-up-a-Down* provide insight into the background of Jarman’s self-imagination as medieval saint. Jarman’s incomplete development of the figure of Lady Angharad precedes his canonization by the Sisters of Perpetual Indulgence and his own experience of HIV/AIDS. The figure of Lady Angharad became recycled in the author’s own autobiographical response to his imagined text. While the cairn did not become an image in *The Garden*, Jarman did set about building stone circles on the coast—circles that remain today as a witness to his vision, at Prospect Cottage.

## Jarman’s Garden as Sacred Archival Site

I decide to leave the BFI library and make my pilgrimage to Dungeness, to Prospect Cottage where public grief is allowed. This space is where the Sisters of Perpetual Indulgence canonized Saint Derek, and their historic ritual is remembered and reiterated through visits such as mine. Through his gardening and artistic practice, Jarman created a space for collective mourning and erotic ceremony. Jarman’s cottage stands in the middle of the garden, the gorse flowering along the path and the horizon extending out in both directions. The plants grow in inauspicious terrain, gravel, salted stones, and sand. In June 1989 Jarman recorded his experience of watching the Kentish coast from the passenger seat of a car: “The world like a medieval miniature or one of the unicorn tapestries in the Cloisters; the gravel path—the road to an earthly paradise, above us a wild sky with a flaming sun in bands of violet, pink, and blue” (Jarman 2018a: 92). The image is precise in its visual detail—the qualities of the colors drawn from close observation of medieval art. The sense of a sacred landscape presents Jarman’s garden as the paradisiacal destination. Daniel O’Quinn (1999: 116) has argued that Jarman’s garden creates a “sacred sodomitical space,” a paradise that has been displaced through institutionalized homophobia. Building on O’Quinn, I contend that in Jarman’s garden there are inscribed queer ritual practices that invite the viewer to participate in the memorialization of those lost to AIDS; through these practices, Jarman’s sacred archive is enlivened and preserved.

In his assemblage paintings, made on-site while planting his garden at Prospect Cottage, Jarman (2018a: 104) explicitly drew on reliquary practices. He describes how he made “a gilded canvas in the manner of a relique: a coffin nail, locks of hair, a broken comb found on my walk yesterday, a diamond, gold ring, ruby blood drop, one lucky stone hanging from a string and a pale pink condom. / Nick, Robert, Terry, Howard, David.” The gilding, jewels, ring, and Christian iconography immediately signal the aesthetics of medieval devotional practice. Jarman’s aesthetic deployment of relic discourse goes beyond the surface. The found stone evokes medieval ideas of sacred geology, evidenced, for example, in one sixth-century reliquary box containing pebbles from Palestine, which was kept for centuries in the Sancta Sanctorum chapel in Rome (Hahn 2017: 25). The blood and lock of hair recall the veneration of broken bodies. The condom acts both as a blood relic and as the sign of martyrdom. The creation of a reliquary sanctifies those lost, invoking their memory as an active presence.

Numerous assemblages illustrate Jarman’s memorializing practice, in which medieval devotional objects are situated in contemporary queer experience. For example, in the work *In the beginning was the word* (1990),^14^ Jarman embeds a whip and two books beneath layers of tar and black paint. As with medieval relics contained in opulent vessels, Jarman’s painting draws attention to its own thickly wrought exterior. The whip evokes both the crucifixion and BDSM, sanctifying queer sex cultures and subverting the queerphobia of the institutional church. The books are illegible beneath paint, creating an artifact designed for touch more than recitation, recalling the talismanic books of premodern nonliterate communities. The work becomes a dual relic: the container both of sacred objects and of the artist himself, traces of whose presence are kept as if in motion across the rippling, dense canvas. The painting refuses framing; the handle of the whip extends beyond the edge of the canvas, demanding to be in open air. The work invites intimacy with the viewer, albeit an intimacy that, in the gallery, is forbidden to touch.

The space at Prospect Cottage offers an embodied experience of engagement with memorializing objects, outside the restrictions of the modern gallery. Grief for those lost to AIDS was at the foundations of Jarman’s herbal garden. He wrote: “My garden is a memorial, each circular bed and dial a true lover’s knot—planted with lavender, helichryssum and santolina. / *Santolina, under the dominion of Mercury resisteth poison, putrefaction, and heals the bites of venomous beasts*” (Jarman 2018a: 55). Jarman’s statement of their role as tokens of mourning is swiftly followed by allusion to premodern medical herbal traditions. Jarman’s direct primary sources for medical herbal lore were early modern works, such as John Gerard’s 1597 *Herball* (Ellis 2014: 388). However, these Renaissance traditions reflect the continuity of medieval understandings of the healing properties of plants. In one fourteenth-century Middle English translation of the influential Latin herbal *On the Properties of Plants*, we learn that lavender “y sodde in water and y dronke ofte is gode to hele the palesy … the smell of this erbe putteþ a way the pestelense” (sodden in water and drunk often is good to heal the palsy … the smell of this herb chases away the plague).^15^ In accordance with Jarman’s vision of the Kentish coastline as a medieval illumination, these premodern texts are drawn into affective, associative relation with the garden. Medieval herb gardens were commonly attached to monasteries, functioning as natural, sacred pharmacies (Dendle and Touwaide 2015). The ability of plants to cure was seen as a reminder of divine grace. Jarman’s garden extends these horticultural traditions, his memorializing practice of queer devotion integrated into a framework of healing. Rather than God’s grace, Jarman’s garden asserts the continued agency of lost queer lives. As relics, his plants evoke both grief and salvation, reimagining the traces of his friends as the source of cure: living, curative ashes.

Jarman (2018a: 87) repeatedly refers to the medieval application of plants as cures for plague: “My blue columbine is in flower … The columbine—aquilegia, the eagle’s foot—a wild flower, has crept into my garden, one of the herbs used against the Black Death in the 14th century.” As Jarman implies, there were many and varied herbal cures for plague in the medieval period. In another early fifteenth-century book of medical treatises, after the scribal phrase “explicit” [here ends], which signals the conclusion of the main text, a medieval reader recorded the following: “For the pestilence A sovereign medicine. Take equal parts fever-few, knapweed, mugwort and marigolds, wash them and stamp them and mix them with old ale and give the sick six spoonfuls to drink at once. And if a man has this medicine in time it will save him from the pestilence.”^16^

The book in which this cure is recorded is luxurious, highly illuminated throughout with images of plants, surgical instruments, and bodies in various states of disease. The definition of a medicine as “sovereign” situates the prescription in its material contexts of wealth and status. The elite medicine is procured from the herb garden—made from native wildflowers and weeds, such as Jarman describes arriving unplanned in his garden. The added recipe reflects the themes of the manuscript, appearing as an extension of the text. The unplanned insertion animates the previous material, situating the lists of herbs and rules for healthy living in their historic context of infectious disease. The manuscript is lent urgency through the awareness of its reception in that context. In medieval books, the curative application of plants appears not as a narrative but as a practical necessity. Jarman does not invite a simplistic, inaccurate equation of the bubonic plague with AIDS, and I do not intend to make that comparison here. However, guided by Jarman, I find myself in the British Library unexpectedly grieving the medieval dead, who sought salvation from ale and marigolds. Jarman’s affective approach to medieval medicine, drawn from his own distinct experience of illness, jolts me out of the complacent, detached relationship of the modern scholar to premodern plague. The handwritten trace, “for the pestilence,” reminds me of the body that sought the cure.

Jarman’s imagination of the garden as a curative memorial was rooted in medieval descriptions of relics and herbals, which were integrated traditions. In the anonymous fourteenth-century dream vision *Pearl*, the dreamer-narrator enters a garden to seek his lost pearl, an allegorized figure of his lost child. As a symbol, the pearl also recalls the parable of Matthew 13:45, in which a pearl stands for the kingdom of heaven. Pearls were frequently used to adorn reliquaries: spiritually resonant objects that reflected and enhanced the viewer’s awareness of their precious contents. Imagining his pearl buried beneath the earth, the grieving narrator declares:

þat spot of spysez mot nedez sprede,/þer such ryches to rot is runne/ … Of goude vche goude is ay bygonne: / So semly a sede moȝt fayly not,/ þat spryngange spychez vp ne sponne / Of þat precios perle wythouten spotte. (Andrew and Waldron 2013: 55, ll. 25–36)

Spices should grow from that spot / where such riches have been left to rot / Of good each good is begun: / So seemly a seed cannot fail, /So that spices will spring up / from that precious pearl without a spot.

The buried pearl is at once an image of rot and renewal: the pearl causes the earth to bring forth marvelous new life. Pearls themselves feature in medieval medical texts: one fourteenth-century herbal recommends as a cure, “Margarita, perle cold and dry.”^17^ The narrator subsequently finds aromatic plants in the garden: cloves, ginger, and the flowering herb gromwell (Andrew and Waldron 2013: 56).

These medicinal herbs reflect the properties of the heavenly pearl, which is itself both spiritually and physically curative. Like a relic, the pearl is figured as sacred remains from which healing plants must grow.

The echoes between the garden in *Pearl* and the garden at Prospect Cottage are strengthened by Jarman’s own allusions to that text. Jarman copied out a passage from a modernized edition of *Pearl* in his project notebook, “A book of colour June 92”:

of Royal pearls a precious piece

There might men by grace have seen

where she as fresh as fleur de lys

came stepping down the sloping green

her gown was glistening white like fleece

with open sides all bound between

by perfect pearls I could not cease

To stare at their unequalled sheen.^18^

Jarman focuses on the moment at which the Pearl-maiden appears to the grieving narrator: his lost pearl resurrected in the figure of a young woman. Here the pearl becomes an articulate relic—speaking through a divine vision to the mourner, brought to life. The imaginative resurrection of the Pearl-maiden combines with the understanding that healing spices will grow from the buried pearl, to give a vivid picture of the lost pearl as a vital subject. The memorializing plants at Prospect Cottage draw on these traditions. As the plants continue to grow, they bear witness to the continued, active presence of those they mourn. Viewing Jarman’s plants through the framework of medieval relic devotion allows their healing properties to be imaginatively united with their position as memorializing objects. The sense of these memorializing plants as curative, sacred, and alive acts as a powerful antidote to the repressive discourses of HIV-positive bodies as polluted symbols of mortality. Invoking medieval traditions in which plants are at once relics and cures, Jarman presents the sacred remains of his lost friends as the essential source of spiritual and bodily healing—relics that retain their power through a combination of communal devotion and the daily routine of gardening.

In the medieval traditions on which Jarman drew, ordinary plants could be considered relic-like. Reflecting on the flower lily of the valley, Jarman (2018a: 45) wrote in 1989: “When Mary wept at the foot of the cross her tears turned into this pure white flower of humility. / The medicinal property of the Virgin’s tears was considered so strong in the Middle Ages that infusions made from them were kept in gold and silver vessels, like the jewelled reliquaries that held fragments of the True Cross.” Jarman’s unreferenced claim has imaginative resonance with medieval spiritual and medical practices. Saints’ tears were treated as relics, typically stored in pewter ampullae: these pilgrims’ flasks were a more practical means of transporting liquid than reliquary boxes (Knight 2016). One Byzantine pilgrim flask in the British Museum features an emblem of Mary at the Holy Sepulchre.^19^ This artifact provides an example of a sacralized vessel that, in function and in form, found echoes in medical flasks of spiritually elevated distillate. In addition, medieval herbals reflect Jarman’s statement that lily of the valley was thought to have medicinal qualities. One fourteenth-century verse describes the medicinal virtues of the lily:

Thre maneres of lelys þer bene … / þat growes in ȝerdes with whit flore / þat is called þe glayglofe poure / And is white so any milke. / Non of þat othere bers non swilke / The maistres sayes with milde molde / In plaisteres for postemes þe leues are gode / If it be menged with swynes grece / It rotes þe postemes withoutene lece / And purges and heles al^20^

There are three types of lilies … / The lily that grows in yards with white flowers / is called the poor glayglofe, / and it is as white as milk. No other lily bears such flowers. / The master says, when mixed with soft dirt, / the leaves are good for plasters for swellings; / if it is mixed with pig’s fat / then it rots the swellings without doubt, / and purges and heals everything.

Whereas Jarman draws on a spiritual imagination that casts the lily of the valley as a Holy Land relic, originating at the foot of the Cross, this anonymous medieval poem records the plant by its vernacular name of *glayglofe* and emphasizes its domestic origin, growing in local fields. Rather than stored in holy crystal, the plant here is mixed with pig fat in order to rot bodily swellings. In one sense this verse markedly contrasts with Jarman’s description. But the appearance of this flower in an herbal rhyme without specific biblical allusion does not undermine Jarman’s sense of the significance of the origin narrative of the Virgin’s tears in medieval medicine. Instead it shows the pervasive and varied absorption of these traditions in medieval English culture. Although association of this flower with the Virgin is not directly referenced here, the wildflower is attributed with a striking potency, with white flowers and properties unlike any other variety of lily. There is a sense of marvel inherent in the application of the plant to swollen limbs. For those who were able to purchase jeweled vessels, medical infusions could be transported in reliquaries; for those who were not, these flowers could be picked from the yard. In the same way, the plants in Jarman’s garden do not require transfusion into crystal or pewter flasks in order to be imaginatively received as curative relics. These objects become sacred through their narrativization as such.

Jarman invokes medieval herbal traditions without proposing the application of herbal cures for HIV/AIDS. At times, Jarman (2018a: 179) viewed his imaginative herbalism with ambivalence, writing: “I plant my herbal garden as a panacea, read up on all the aches and pains that plants will cure—and know they are not going to help. The garden as pharmacopoeia has failed.”

In a sense, for Jarman these herbs were faux relics, claiming and failing to heal—and yet these healing narratives remain a constant presence in his journals. Jarman’s herbalism is not merely a placebo. The image of the fake reliquary offers a model for understanding Jarman’s continued investment in medieval medical herbal traditions, in the knowledge that these plants will not cure him of AIDS. In March 1990 Jarman visited a British Museum exhibition of fakes in their collection. To his initial dismay he found a pearl reliquary, noting: “oh heavens, my little twelfth century pearl reliquary from Scotland is there, the most precious object in the museum. But it is so exquisite, being ‘fake’ cannot diminish it” (Jarman 2018a: 253). The reliquary is not a postmedieval imitation but was included in the exhibition as an example of medieval devotion to fake relics. Jarman swiftly assimilates the knowledge of the inauthenticity of his most beloved artifact into his affective response, allowing the object to remain elevated. Jarman’s continued veneration of the relic, after its official disqualification as such, indicates his affective proximity to traditions of medieval relic devotion, in which the audience were given a crucial role in authenticating or discrediting the venerated object. Relic discourse is ostensibly undermined by the knowledge of the inauthenticity of the venerated object—yet as Jarman shows, the continuation of a ritual enables the continued existence of the relic.

In medieval and queer devotion alike, faux relics stay real through reiterative performance. These objects collapse the official logic of relics as non-substitutable: they are both irreplaceable and inauthentic. Built around these traces of the dead, the queer community preserves saints through practices of memorialization that themselves depend on the preservation of queer spaces: like Prospect Cottage, a saint’s garden.

## AIDS in the Art Archive

To the extent that I came to the Jarman archives in search of the unique, non-reproducible, and irreplaceable work of art, the location of Tate Britain contributed to the canonization of Jarman as artist. The elite repository confirmed the authenticity of its contents. But to the extent that I came to the archives and encountered the haphazard traces of a queer life during the AIDS crisis, that same canonizing repository failed to accommodate my reaction, integrating its traumatic contents into the idea of the artist, held apart from the community.

In 1993 the Department of Health, under the direction of Virginia Bottomley, secretary of state for health from 1992 to 1995, proposed the closure of St. Bartholomew’s hospital, then one of only two dedicated HIV/AIDS research and treatment centers in the UK. Near the end of his life at this point, Jarman—staying in the Colston ward of St. Bartholomew’s hospital—wrote a letter to the *Independent*, seeking to raise public opposition to the proposed closure. Jarman (1993b) situates his experience on the wards in direct relation to the medieval history of St. Bartholomew’s as, in his words: “the world’s oldest hospital, founded in the 11th century by the monk Rahere. He fell ill with a quartan fever on a pilgrimage to Rome and was commanded by Saint Bartholomew in a dream to build a hospital. When he returned to England, he was given the gift of this land in ‘Smoothfield’ by the King.”

The hospital is presented as a sacred space, the consequence of a divine vision. The land retains the trace of a vision received in a time of sickness: the foundation of the hospital is attributed to the inspiration of a fever, placing the experience of illness at the center of the medical establishment. The claim for St. Bartholomew’s to be the “world’s oldest hospital” is spurious, but the rhetorical hyperbole is effective. Jarman (1993b) goes on: “I … read in the pavilion to the sound of the fountain, before retiring to the hospital church of Saint Bartholomew, which itself is cool and filled with the peace of time.” In his draft notes for this letter, Jarman writes, “the church hospital is a parish.”^21^ The erasure in his notes becomes a composite category in his public letter, the binary between hospital and church collapsed through the invocation of the sacred origin narrative of the space.

In his hospital notes, Jarman presents the preservation of St. Bartholomew’s as an act of saint worship. Addressing the government in verse, he writes in shaking handwriting, on loose lined sheets:

I feel you might

make the right decision

and kep [*sic*] barts

the gift of a saint

a vision is too precious

will your vision of a

restructured Health Service

which no one grudges

you last as long

800 years of our

National history^22^

Viewing the hospital through its medieval history places Thatcherite policies in perspective—as a transient moment in comparison to the longevity of medieval saint worship. Rather than comparing the cuts to the 1948 terms of the foundation of the National Health Service, Jarman draws on medieval discourses that present health care in relation to a different service: the church liturgy. Inhabiting the hospital becomes a pious act, presenting Jarman’s position on the ward as an active rather than a passive relationship to the space. The treatment of AIDS patients in St. Bartholomew’s Hospital becomes divinely mandated, overriding the edicts of the Health Department. The hospital witnesses its position as the reliquary of St. Bartholomew’s vision through continuing to function actively as a space of care. Although Jarman uses the language of medieval medicine as an alternative to oppressive contemporary discourses of HIV/AIDS, he does not establish a simplistic binary between a transcendent medieval and a repressive present. Instead, here he uses the medieval to give a sacred lineage to the modern clinical space that he inhabited, which becomes a space of veneration. Closing the hospital becomes iconoclasm.

In the Tate archives, I found myself thrown by the encounter with Jarman’s present-tense voice from the hospital bed, speaking in justifiable rage: “why am I stuck in this ward when money is available but frozen to build a ward for HIV patients.”^23^ The Colston ward, designated for palliative care, was transformed into an overspill ward for AIDS patients. As Ann Cvetkovich (2003: 249) writes, “While any archive is haunted by the spectre of death, its literal and traumatic presence in any AIDS collection serves as a vivid reminder that archives can be motivated by emotional rather than intellectual needs.” Jarman’s archives are not an AIDS collection*—*yet the realities of his experience of illness permeate his art and writing. The preservation of these papers in the Tate collection positions them as the marvelous traces of a canonical artist. The repository elevates and conserves—sacralizes—its contents. But the encounter with the unique work of art also brought me into contact with Jarman’s experience as a member of the queer community during the AIDS crisis. The encounter with these traces of the dead collided with the experience of artistic aura—unable to interpret and refusing to aestheticize the suffering of the recent past, I was exhausted at the end of each day. The prestige of art amidst the traumatic records of defunded hospitals began to sicken me; I could feel the pressure to integrate these materials into a cohesive narrative of oppressed genius whose suffering is offset by artistic production. The establishment mode of veneration that removes the artist from their community is still more jarring when the state has been the source of communal death. Resisting that pressure, I aim to recognize the simultaneous existence of distinct elements in these collections. Rather than one archive, I experienced Jarman’s archives as being plural even within one repository and as multiplying across their different archival sites.

## Queer Afterlives of Jarman’s Archives

In one abandoned plan for his remains, Jarman (2019a: 105) wrote: “I’ll be cremated and have Christopher mix the ashes with black paint and paint five canvases which I’ll have signed—it’ll be my last artwork.” The authorial signature beneath the ashes recalls the self-authenticating quality of relics, which speak from their position as marvelous ashes, traces of saints who continue to exert their divine influence. The affective responses that Jarman’s archives demand—rage, grief—were uneasily accommodated in the conventional spaces at Tate Britain and the BFI, where I was unwilling to cry, unable to share pictures with friends so that they could join me in mourning. This is the closest I can come to a communal experience—inviting you, reader, to grieve with me here.

**Figure 2:**
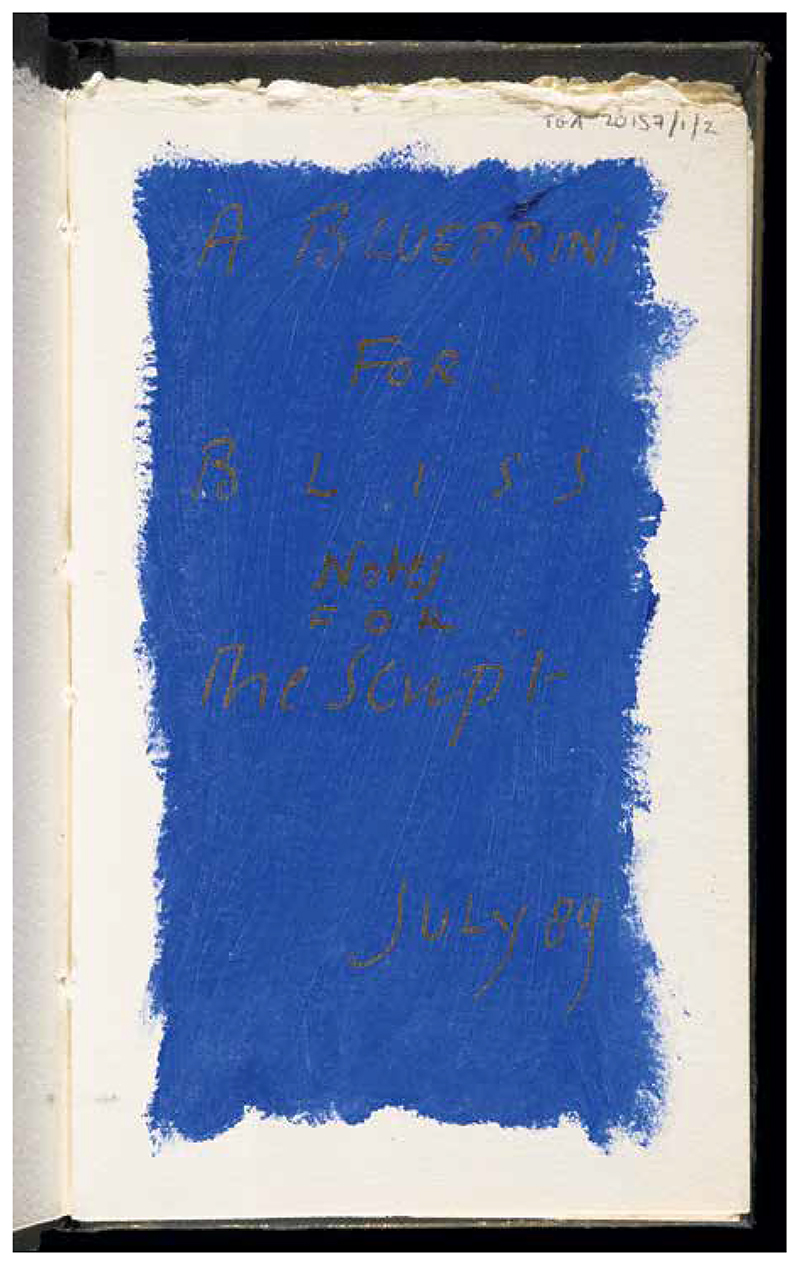
“A Blueprint for Bliss: Notes for the Script 1989,” Derek Jarman, TGA 20157/1/2, Tate Britain. © Photo © Tate. Courtesy Keith Collins Will Trust.

At my desk in the Tate, I take a tissue-wrapped book from a brown cardboard box, carefully remove the shroud with gloved hands, and lay the book on a cushion. It is an early project book for Jarman’s final film, *Blue* (1993a). There is a single vein in the stone on the cover. I stroke it with my plastic-sheathed fingertip. Alone with hand-painted pages, I experience an intimate relationship with the artwork. It is both a critical and a ritualistic encounter. The trappings of the archive become cultic. The room is quiet—an invigilator watches me work from behind a glass screen. Jarman inscribed in gold text over a bright front leaf of ultramarine blue: “A Blueprint for BLISS / Notes for the Script / July 89.”^24^ In content as well as form, the book is a devotional object: the black cover of the notebook is hand decorated with gilt paint, with a dark blue stone affixed to the center of the cover. Written in gilded letters over blue paint in which the brushstrokes remain visible, the page emphasizes its status as devotional artifact (fig. 2). On the first page, Jarman writes that “Blueprint for Bliss addresses the audience in the first person intimately.”^25^ Both the form and content of “Blueprint” invite an affective mode of engagement on the part of the reader. The notebook is presented as a hagiographic text, a dialogue between devotee and saint: describing the painter Yves Klein’s desire for the healing body of the medieval Saint Rita of Cascia. Jarman writes:

At the far edge of the known

WORLD is the shrine to RITA

Where all at the end of the line call, for she is the saint of the LOST CAUSE. The saint of all those at their wites end, who are hedged in and trapped by the FACTS that clutter the world.^26^

This text was reprinted in *Chroma* (Jarman 2019b: 90) and became part of the voice-over of *Blue*, but there is an additional significance to the passage as it is represented in this notebook. The book is small—in its size, as well as in its jeweled and gilded decorations, it recalls the intimacy of medieval devotional books, designed to be portable, pocket-sized companions for prayer and contemplation. In *Blue*, poetry, quotations, and extracts of Jarman’s hospital diaries form a soundtrack heard over a screen onto which a single, solid color is projected, International Klein Blue (IKB), refusing straightforward cinematic representation (Khalip 2010). The film refuses to leave the viewer with an external image to hold. Jarman’s experience of his own eyesight deteriorating from cytomegalovirus (CMV), an AIDS-related condition, is central to both the narrative and form of the film: as the voice-over narrates Jarman’s sensation of pulsating blue flashes at the eye clinic, the screen pulses blue. The project book, created in the earlier stages of Jarman’s condition, remains a definitively visual and tactile object, while articulating and anticipating a nonvisual encounter with “the immaterial the void an architecture of air.”^27^ Presented as an address to St. Rita, the notebook reaches beyond the material toward dialogue with the immaterial. Jarman’s project book draws on the duality of medieval relics, which oscillate between transcendence and earthly form. An audiovisual event that took place as a precursor to *Blue* shows how relic devotion shaped Jarman’s construction of the project. On January 6, 1991, at a prescreening event for *The Garden* at the London Lumiere, slides of an Yves Klein painting were projected onto the screen, to musical accompaniment conducted by Simon Turner, the composer of the final soundtrack for *Blue* (O’Pray 1996: 201). At the end of the event, the child actor Jody Graber went into the audience to distribute blue-and-gold–painted stones. These stones—like that which Jarman affixed to his “Blueprint” journal—recall the tokens received at a shrine, pilgrimage souvenirs.

Jarman’s “Blueprint” notebook offers an alternative route into understanding the aural experience of *Blue*. Placing the film in dialogue with its notebook, *Blue* appears as an audiovisual response to material cultures of devotion. As a color, blue has a long symbolic tradition in visual representation; it is seen, for example, in medieval images of the Virgin’s robes. While exploring the visionary potential of the immaterial, *Blue* draws on Jarman’s pre-CMV engagement with IKB as a painterly medium, a material pigment. The fragility of the “Blueprint” notebook becomes the fragility of sight in the film. *Blue* becomes a vision experienced at a shrine: sound emanating, articulating a deep yearning for the absent image.

In withdrawing the reproducible image, *Blue* aims to transfigure the medium of film: Benjamin’s (2002: 109) original example of an art form whose “character is entirely determined by its reproducibility.” Listen to the close of Jarman’s film, his last: “I place a delphinium, blue, upon your grave.” Jarman’s blue flower floats into the mind not as technological reproduction, but in the imagination. In 1936 Benjamin wrote of cinema that “the sight of immediate reality has become the ‘blue flower’ in the land of technology” (Hansen 1987: 204). Benjamin’s metaphor evokes the cinematic spectator’s impossible desire for a tangible object. I find an irresistible resonance between Jarman’s “delphinium, blue” and Benjamin’s “blue flower.” Expressed in a common language, both invoke nature in order to image aural experience, situated in the cinema. In Benjamin’s analysis, the impossible blue flower resists the desensitizing operation of the cameraman, who is paired with the surgeon: the “technoaesthetics” of cinema contextualized in relation to medical anaesthetics (Buck-Morss 1992: 18). In Jarman’s film, the blue flower resists the anaesthetized response of the state to AIDS patients, figuring grief where care has been denied. Through visualization of Jarman’s delphinium, an embodied ritual comes into existence.

Roger Hallas (2009: 33) argues that *Blue* “implicates the spectator’s own body in the process of bearing witness to AIDS.” To me, *Blue* inscribes not only an act of witness but also an act of devotion. Through contemplation, *Blue* is performed in the body. Washed with color reflected from the screen, I experience the sensation of Jarman’s voice, appearing in the soundtrack amidst the ventriloquism of actors John Quentin and Nigel Terry. The magnetic tape of the audio recording becomes a reliquary, preserving an authentic fragment of Jarman. Instead of being reproduced, *Blue* is recirculated like a relic: at galleries and cinemas, on private laptop and phone screens, making these objects into sacred vessels for the duration of the vision. Jarman’s relics are self-authenticating objects that speak to their devotees, relying on the community to receive and reiterate their vision.

In “A Blueprint for BLISS,” the image of the subterranean, sacred vessel returns. On a page in gilded letters over blue paint, in Yves’s final prayer to St. Rita, Jarman writes of a

solemn landscape of dark waters

washing the isle of the dead

Where Pearl fishers

embrace

amphora

…

Lost boys!

sleep forever

in a dear embrace

salt lips touching

in submarine gardens.^28^

The poem is presented as a direct address to the lost boys. Inscribed in devotional style, the poem in this notebook speaks in apostrophe from within its tissue wrappings, its cushion. The book becomes a sacred container for the submarine garden of the text: situating the sleeping lovers in their submerged, wrecked shrine. Jarman’s poem exists in multiple iterations, both inside and outside the archives. In a later notebook, dated 1992, also entitled “Blueprint,” Jarman recorded the same text under the title “Two Queers Dreaming.”^29^ The variant transcription of the text, repeatedly copied out by its author by hand, echoes medieval practices of manuscript composition, in which scribes repetitively rewrote and collected texts to keep them in circulation. The title given to the lyric in Jarman’s 1992 notebook repositions the poem as the vision of the sleeping boys—who become not merely the subject of the vision but also the visionary dreamers. The sleeping queers find themselves within their own dream, prefiguring their own deaths, but also returning the inhabitants of the “isle of the dead” to a state of unconscious agency. There is a notable difference between this poem in the 1989 and 1992 notebooks—in the earlier version, the text is quasi-liturgical, a prayer at a shrine; in the later version, the text is a dream vision received from within the submarine shrine itself. A version of the same text was printed in *Chroma*, with the exclamation mark removed, creating ambiguity as to whether the lines are addressed to—or describing—the lost boys (Jarman 2019b: 98). Circulating between Jarman’s notebooks and works, the poem is itself treated like a relic, a fragment that enlivens its surroundings.

In being enshrined at the Tate and the BFI, Jarman’s archives have been made available to the public—but they are also removed from touch, inaccessible to those for whom institutional spaces prove unassailable barriers. The ambivalent containment of these archives confirms that their real home will remain Dungeness: the boxes are homesick for Keith, their true custodian, and for the paintings and books on Jarman’s walls. My encounter with these collections speaks to what is lost when a queer artist is canonized by the mainstream establishment, as well as what is gained in cultural and financial terms. The institutional canonization of Jarman grants his works the security of the temperature-controlled vault. Although the position of these materials in the quiet repository heightens their associations of grief, the archive is a space of futurity, of survival. Looking to the medieval past for an alternative language of communal mourning and veneration, Jarman asserted that the conservatism and trauma through which he lived was impermanent. Jarman’s archives explicitly speak to the queers of the future, those who “will come again” in all senses.^30^ Jarman’s archives inscribe their own ceremonies of veneration—if they were able to leave the repository, they would do so in a procession, paraded and erotically stroked. These queer remains live on through the queers who remain.

## Figures and Tables

**Figure 1:**
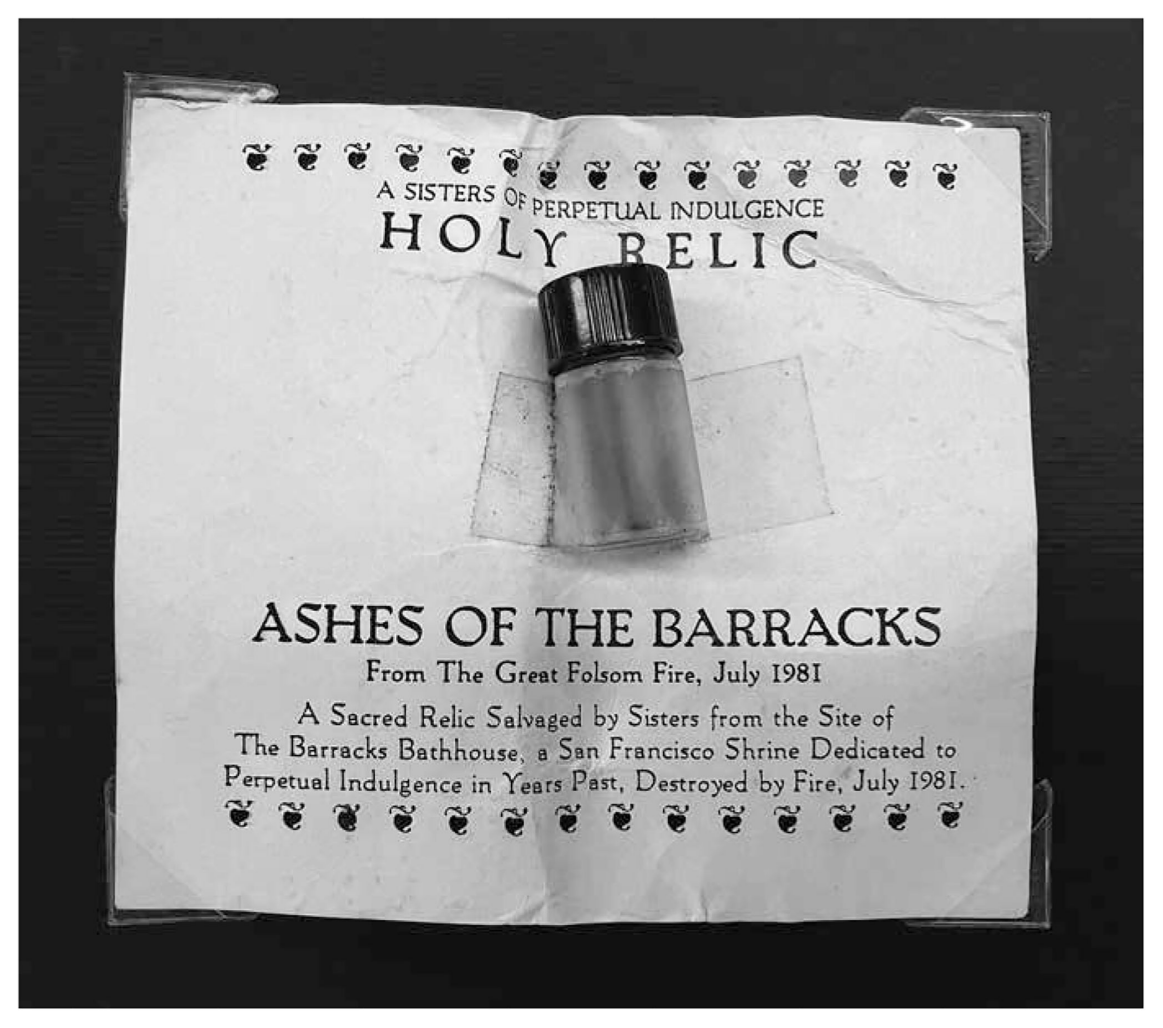
A sacred relic from the site of the Barracks Bathhouse. Papers of Fabian LoSchiavo. Courtesy Australian Queer Archives.

## Footnotes

1 Jarman, script for *Bob up a Down*, February 24, 1981, box 42, item 1, Derek Jarman II Special Collections, BFI National Archive, London (hereafter Special Collections).

2 Jarman, script for *Bob up a Down*, February 24, 1981, box 42, item 1, Special Collections.

3 Jarman, selection of pink booklets, box 42, item 6, Special Collections.

4 Jarman, “Bob up a down Derek Jarman Tim Sullivan Oct 1983,” box 42, item 2, Special Collections.

5 Jarman, selection of pink booklets, box 42, item 6, Special Collections.

6 Jarman, selection of pink booklets, box 42, item 6, Special Collections.

7 Jarman, “Bob up a down Derek Jarman Tim Sullivan Oct 1983,” box 42, item 2, Special Collections.

8 All modern English translations are my own.

9 Jarman, selection of pink booklets, box 42, item 6, Special Collections.

10 Jarman, selection of pink booklets, box 42, item 6, Special Collections.

11 Jarman, selection of pink booklets, box 42, item 6, Special Collections.

12 Jarman, selection of pink booklets, box 42, item 6, Special Collections.

13 Jarman, “In Borrowed Time” (poem), TGA 2021/3 Jarman, Tate.

14 Jarman, *In the beginning was the word*, 1990, tar and mixed media on canvas, 56.5 × 38.4 cm, Amanda Wilkinson Gallery Online, https://amandawilkinsongallery.com/exhibitions/133/works/image1255/ (accessed July 20, 2022).

15 Jarman, Sloane MS 5, fols. 32r-v, British Library, London.

16 Jarman, additional MS 29301, fol. 95v, British Library, London.

17 Jarman, Wellcome MS 397, fol. 81r, Wellcome Library, London.

18 Jarman, “A Book of Colour June 92,” TGA 20157/1/5, Tate TGA.

19 Ampulla. Pewter, 11th–13th century, Byzantium, Museum no. 1901,0529.24. British Museum, London. https://www.britishmuseum.org/collection/object/H_1902-0529-24.

20 Jarman, additional MS 17866, fol. 11r, British Library, London.

21 Jarman, folder, “Bedroom,” clear plastic folder re: “Death in Venice,” 19 pp., Tate.

22 Jarman, folder, “Bedroom,” clear plastic folder re: “Death in Venice,” 19 pp., Tate.

23 Jarman, folder, “Bedroom,” clear plastic folder re: “Death in Venice,” 19 pp., Tate.

24 Jarman, “A Blueprint for Bliss Notes for the Script 1989,” TGA 20157/1/2, Tate Britain, London (hereafter Tate).

25 Jarman, “A Blueprint for Bliss Notes for the Script 1989,” TGA 20157/1/2, Tate.

26 Jarman, “A Blueprint for Bliss Notes for the Script 1989,” TGA 20157/1/2, Tate.

27 Jarman, “A Blueprint for Bliss Notes for the Script 1989,” TGA 20157/1/2, Tate.

28 Jarman, “A Blueprint for Bliss Notes for the Script 1989,” TGA 20157/1/2, Tate.

29 Jarman, “Blueprint 1992,” TGA 20157/1/4, Tate.

30 Jarman, folder, “Attic,” Basildon Bond writing pad: “Death in Venice Poems,” 18 pp., TGA 2021/3, Tate.

## References

[R1] Andrew Malcolm, Waldron Ronald (2013). The Poems of the Pearl Manuscript in Modern English Prose Translation: Pearl, Cleanness, Patience, Sir Gawain, and the Green Knight.

[R2] Benjamin Walter, Jephcott Edmund, Zohn Harry, Eiland Howard, Jennings Michael W (2002). Walter Benjamin: Selected Writings.

[R3] Buck-Morss Susan (1992). Aesthetics and Anaesthetics: Walter Benjamin’s Artwork Essay Reconsidered. October.

[R4] Bynum Caroline Walker (1991). Fragmentation and Redemption: Essays on Gender and the Human Body in Medieval Religion.

[R5] Cvetkovich Ann (2003). An Archive of Feelings: Trauma, Sexuality, and Lesbian Public Cultures.

[R6] Davies RT (1966). Medieval English Lyrics: A Critical Anthology.

[R7] Dendle Peter, Touwaide Alain (2015). Health and Healing from the Medieval Garden.

[R8] Dinshaw Carolyn (1999). Getting Medieval: Sexualities and Communities, Pre- and Post-modern.

[R9] Ellis Jim (2009). Derek Jarman’s Angelic Conversations.

[R10] Ellis Jim (2014). Renaissance Things: Objects, Ethics, and Temporalities in Derek Jarman’s Caravaggio (1986) and Modern Nature (1991). Shakespeare Bulletin.

[R11] George Jodi-Anne, Newland Paul, Hoyle Brian (2019). British Art Cinema.

[R12] Hahn Cynthia (2017). The Reliquary Effect.

[R13] Halberstam Jack (2005). In a Queer Time and Place: Transgender Bodies, Subcultural Lives.

[R14] Hallas Roger (2009). Reframing Bodies: AIDS, Bearing Witness, and the Queer Moving Image.

[R15] Hansen Miriam (1987). Benjamin, Cinema, and Experience: ‘The Blue Flower in the Land of Technology’. New German Critique.

[R16] Horrox Rosemary (1995). The Black Death.

[R17] Jarman Derek (1990). The Garden.

[R18] Jarman Derek (1993a). Blue.

[R19] Jarman Derek (1993b). Letter: Why Shutting Bart’s Would Be a Crime. Independent.

[R20] Jarman Derek (2018a). Modern Nature: The Journals of Derek Jarman, 1989–1990.

[R21] Jarman Derek, Christie Michael (2019a). At Your Own Risk.

[R22] Jarman Derek (2019b). Chroma: A Book of Colour—June ‘93.

[R23] Windeatt Barry, Julian of Norwich (2015). Julian of Norwich: Revelations of Divine Love: The Short Text and the Long Text.

[R24] Khalip Jacques (2010). ‘The Archaeology of Sound’: Derek Jarman’s *Blue* and Queer Audiovisuality in the Time of AIDS. Differences.

[R25] Knight Kimberley-Joy (2016). Droplets of Heaven: Tear Relics in the High and Later Middle Ages. Medieval Journal.

[R26] Kumbier Alana (2014). Ephemeral Material: Queering the Archive.

[R27] Mills Robert (2018). Derek Jarman’s Medieval Modern.

[R28] Mills Robert, Coomasaru E, Deichert T (2022). Imagining the Apocalypse: Art and the End Times.

[R29] Myerson EK (2023). Derek Jarman’s Medieval Blood: Queer Devotion, Affective Medicine, and the AIDS Crisis. Postmedieval.

[R30] O’Pray Michael (1996). Derek Jarman: Dreams of England.

[R31] O’Quinn Daniel (1999). Gardening, History, and the Escape from Time: Derek Jarman’s ‘Modern Nature’.

[R32] Peake Tony (1999). Derek Jarman.

[R33] Russell Catherine (2018). Archiveology: Walter Benjamin and Archival Film Practices.

[R34] Salih Sarah, Salih Sarah, Baker Denise Nowakowski (2009). Julian of Norwich’s Legacy: Medieval Mysticism and Post-medieval Reception.

[R35] Watney Simon, Crimp Douglas (1988). AIDS: Cultural Analysis, Cultural Activism.

